# Haemophilus parainfluenzae Pyogenic Liver Abscess Associated With Cholangiocarcinoma

**DOI:** 10.7759/cureus.22501

**Published:** 2022-02-22

**Authors:** Mathew C Finniss, Lamis Ibrahim

**Affiliations:** 1 Department of Infectious Diseases, East Tennessee State University Quillen College of Medicine, Johnson City, USA

**Keywords:** cholangiocarcinoma, hepatobiliary infection, liver abscess, pyogenic, haemophilus parainfluenzae

## Abstract

*Haemophilus parainfluenzae* (*H. parainfluenzae*) is a commensal organism of the gastrointestinal tract. It rarely causes hepatobiliary infections; however, in the presence of underlying inflammation, immunosuppression, or malignancy, it can cause hepatobiliary infection via an ascending route. Herein, we report a case of pyogenic liver abscess secondary to *H. parainfluenzae* associated with cholangiocarcinoma, which was treated with ceftriaxone and metronidazole.

## Introduction

*Haemophilus parainfluenzae* is known to cause meningitis, epiglottitis, pneumonia, bacteremia, endocarditis, and a wide variety of other infections [1-4]. *H. parainfluenzae* is a commensal organism of the gastrointestinal tract but rarely causes hepatobiliary infections [3,4]. To date, only five cases of *H. parainfluenzae* pyogenic liver abscesses and two cases of *H. parainfluenzae* biliary tract infection have been reported [3].

The most likely mechanism of hepatobiliary infection by *H. parainfluenzae* is via an ascending route [1,2,4,5]. The abundance of factor V in the gastrointestinal tract and the similarity of *H. parainfluenza*e outer-membrane proteins to that of other enteric flora allow for the growth and adhesion of *H. parainfluenzae* to the intestinal mucosa [5]. Concomitant inflammation, immunosuppression, or malignancy can lead to *H. parainfluenzae* overgrowth and ascending biliary tract infection [5]. Herein, we report a case of *H. parainfluenzae* pyogenic liver abscess associated with cholangiocarcinoma.

## Case presentation

A 56-year-old female patient with a past medical history of rheumatoid arthritis and hypertension presented to the hospital with fever and abdominal pain. On admission, she had a temperature of 98.1°F, a respiratory rate of 20 breaths per minute with an oxygen saturation of 85% on room air, a heart rate of 114 beats per minute, and a blood pressure of 143/65 mmHg. Initial laboratory values were significant for anemia, leukocytosis, elevated alkaline phosphatase, elevated total bilirubin, and transaminitis (Table 1).

**Table 1:** Laboratory values of the patient.

	Value	Reference range	Units
Hemoglobin	11.5	13.5-17.5	g/dL
White blood cell	17.5	3.5-10.5	K/uL
Creatinine	0.9	0.9-1.3	mg/dL
Alkaline phosphatase	176	34-104	U/L
Total bilirubin	0.9	0.3-1.2	mg/dL
Alanine aminotransferase	68	17-63	U/L
Aspartate aminotransferase	112	15-41	U/L

Computed tomography (CT) of the abdomen/pelvis with and without contrast was significant for acute sigmoid diverticulitis complicated by a pericolonic sigmoid abscess, a large multiloculated liver abscess, and retroperitoneal adenopathy. She underwent CT-guided percutaneous drainage of the liver abscess and fluid cultures were positive for *H. parainfluenzae*. Magnetic resonance cholangiopancreatography was done to evaluate the relation of the gallbladder and biliary anatomy to the liver abscess and demonstrated ruptured cholecystitis communicating with the intrahepatic abscess as well as narrowing of the common bile duct. She underwent open cholecystectomy and drainage of the intrahepatic abscess. Intra-operative cultures were negative, but pathology was positive for poorly differentiated cholangiocarcinoma. Blood cultures remained negative throughout and she was successfully treated with a four-week course of ceftriaxone and metronidazole.

## Discussion

*H. parainfluenzae* colonizes the gastrointestinal tract and has been detected in up to 20% of stool samples, but rarely causes hepatobiliary infections [5]. A literature review published by Athreya et al. identified five cases of *H. parainfluenzae* pyogenic liver abscess while a separate report from Friedl et al. identified two cases of *H. parainfluenzae* biliary tract infection [3,5]. Although considered rare, an earlier commentary by Pierard et al. suggests that *H. parainfluenzae* hepatobiliary infections may be under-recognized. A retrospective analysis of 45 bile specimens cultured on blood agar supplemented with factor V isolated *H. parainfluenzae* in 6.6 % of samples [6]. Therefore, plating bile specimens on a medium supplemented with factor V may increase the detection of *H. parainfluenzae* in hepatobiliary infections.

The exact mechanism of *H. parainfluenzae* hepatobiliary infections is unknown. *H. parainfluenzae* is known to colonize the duodenum and *H. parainfluenzae* overgrowth in the presence of underlying hepatobiliary disease likely results in an ascending infection [1,2,4,5]. This proposed mechanism is supported by the presence of factor V, a growth factor for *H. parainfluenzae*, in the intestinal tract and the adhesion of *H. parainfluenzae* outer-membrane proteins to the intestinal tract mucosa [4,5]. None of the seven cases of *H. parainfluenzae* hepatobiliary infections report concurrent *H. parainfluenzae* bacteremia, which is another reason to favor local infection over hematologic seeding [3-5].

Of the previously reported *H. parainfluenzae* pyogenic liver abscesses, the most common presenting features were fever and abdominal pain [3]. More than half of the patients were immunosuppressed, with a chronic granulomatous disease, chronic cholecystitis, and cholangiocarcinoma reported as underlying immunocompromising conditions [3]. *H. parainfluenzae* isolates were ampicillin susceptible in most cases and were successfully treated with ampicillin, cephalosporins, or fluoroquinolones [3,5].

Herein, we report a case of *H. parainfluenzae* pyogenic liver abscess, a relatively rare and possibly under-recognized hepatobiliary infection. Seven cases of *H. parainfluenzae* hepatobiliary infection (five pyogenic liver abscesses and two biliary tract infections) have been previously reported in the literature [3,4]. Like other reported cases, our patient presented with fever and abdominal pain. Our patient did not have concurrent bacteremia, supporting the hypothesis of localized ascending infection. Unfortunately, no susceptibility testing was performed on our *H. parainfluenzae* isolate; however, our patient was treated successfully with a four-week course of ceftriaxone and metronidazole. Our case was also associated with cholangiocarcinoma, which has been reported in one previous case of *H. parainfluenzae* pyogenic liver abscess [5].

## Conclusions

*H. parainfluenzae* colonizes the gastrointestinal tract but is an uncommon cause of infection, except when there is an underlying hepatobiliary disease. Previously reported cases of *H. parainfluenzae* pyogenic liver abscess have been associated with chronic granulomatous disease, chronic cholecystitis, and cholangiocarcinoma. Our case is only the second report of *H. parainfluenzae* pyogenic liver abscess associated with cholangiocarcinoma. Isolation of *H. parainfluenzae* from a pyogenic liver abscess should prompt further investigation of predisposing conditions, such as cholangiocarcinoma.
